# Genome-based analyses to learn from and about **Paenibacillus sonchi* genomovar Riograndensis SBR5T*

**DOI:** 10.1590/1678-4685-GMB-2023-0115

**Published:** 2024-01-05

**Authors:** Volker F. Wendisch, Luciana F. Brito, Luciane M.P. Passaglia

**Affiliations:** 1 Bielefeld University, Faculty of Biology, Genetics of Prokaryotes, Bielefeld, Germany.; 2 Bielefeld University, Center for Biotechnology (CeBiTec), Bielefeld, Germany.; 3 Norwegian University of Science and Technology, Department of Biotechnology and Food Science, Trondheim, Norway.; 4 Universidade Federal do Rio Grande do Sul (UFRGS), Programa de Pós-Graduação em Genética e Biologia Molecular, Instituto de Biociências, Departamento de Genética, Porto Alegre, RS, Brazil.

**Keywords:** Plant growth-promoting bacterium, Paenibacillus sonchi, genome sequencing, genetic tools, nitrogenases enzymes

## Abstract

*Paenibacillus sonchi* genomovar Riograndensis SBR5T is a plant growth-promoting rhizobacterium (PGPR) isolated in the Brazilian state of Rio Grande do Sul from the rhizosphere of *Triticum aestivum.* It fixes nitrogen, produces siderophores as well as the phytohormone indole-3-acetic acid, solubilizes phosphate and displays antagonist activity against *Listeria monocytogenes* and *Pectobacterium carotovorum.* Comprehensive omics analysis and the development of genetic tools are key to characterizing and engineering such non-model microorganisms. Therefore, the complete genome of SBR5T was sequenced, and shown to encode 6,705 proteins, 87 tRNAs, and 27 rRNAs and it enabled a landscape transcriptome analysis that unveiled conserved transcriptional and translational patterns and characterized operon structures and riboswitches. The pangenome of *P. sonchi* species is open with a stable core pangenome. At the same time, the analysis of genes coding for nitrogenases revealed that the trait of nitrogen fixation is sparse within the Paenibacillaceae family and the presence of Fe-only nitrogenase in the *P. sonchi* group was exclusive to SBR5T. The development of genetic tools for SBR5T enabled genetic transformation, plasmid construction for constitutive and inducible gene expression, and gene repression using the CRISPRi system. Altogether, the work with *P. sonchi* can guide the study of non-model bacteria with economic potential.

## Introduction

The genus *Paenibacillus* comprises Gram-positive facultative anaerobic, endospore-forming bacteria. They occur throughout nature, e.g., in water, soil, rhizosphere, and insect larvae. While *P. larvae* causes American foulbrood in honeybee larvae (Daisley et al., 2023), *Paenibacillus* sp. str. FPU-7 is known for its chitinolytic activity of insoluble shrimp chitin flakes (Itoh, 2021). Several *Paenibacillus* species belong to the microbiomes of agriculturally important crops, such as the diazotrophic plant growth-promoting *Paenibacillus polymyxa* that is used as an inoculant in agriculture (Beneduzi et al., 2012; Souza et al., 2015; Langendries and Goormachtig, 2021. This review focuses on the diazotrophic plant growth-promoting *Paenibacillus sonchi* genomovar Riograndensis strain SBR5T. Although the organism displays important growth-promoting traits, its full characterization remains unfinished. For the characterization and engineering of this non-model bacterium, genetic tools, and omics databases had to be developed. Examples of the technological developments and the insights gained are given as well as a perspective on how this knowledge can be leveraged to deepen our understanding of *P. sonchi* with regard to its physiology, genetics, evolution, and application as plant growth-promoting rhizobacterium (PGPR). Analyses performed on *P. sonchi* can also serve as a guide for work involving bacteria isolated from the environment that are difficult to handle.

## *P. sonchi* genomovar Riograndensis SBR5T: isolation, (pan)genome and RNA landscape

*Paenibacillus* strain SBR5T was isolated in the Brazilian state of Rio Grande do Sul from the rhizosphere of *Triticum aestivum* by the laboratory of Luciane Passaglia at UFRGS and named *P. riograndensis* (Beneduzi et al., 2008; Beneduzi *et al.*, 2010). Later, by using genome-based metrics and phylogenetic analyses the strain was shown to be a genomovar of *Paenibacillus sonchi* X19-5T (Sant’Anna et al., 2017), which was described shortly before strain SBR5T (Hong et al., 2009). The physiological characteristics of this facultatively anaerobic, endospore-forming bacterium comprise menaquinone MK-7 as major respiratory quinone, anteiso-C15:0 as major fatty acid, utilization of starch, production of dihydroxyacetone and catalase (Beneduzi *et al.*, 2010). 

*P. sonchi* genomovar Riograndensis SBR5T is a PGPR and was demonstrated to improve the growth of wheat in greenhouse conditions (Beneduzi et al., 2008; Campos et al. 2015). Its plant growth-promoting activities are associated with its ability to fix nitrogen, solubilize phosphate and produce siderophores and the phytohormone indole-3-acetic acid. It also displays antagonist activity against *Listeria monocytogenes* and *Pectobacterium carotovorum* (Bach et al., 2016b).

After the isolation of a new bacterium, the determination of its complete genome sequence provides the first systems-level insight into its lifestyle and provides the basis for further omics-based technologies for systems-level characterization and engineering. A draft genome sequence of SBR5T comprised of 2,276 contigs identified the absence of plasmids, a GC content of 55.1%, and 7,467 open reading frames (Beneduzi et al., 2011). Later, the complete genome sequence was obtained by sequencing two shotgun Paired-End and Mate-Pair libraries followed by joining all contigs, closing gaps, and resolving SNPs in repetitive regions (Brito et al., 2015). While the GC content was confirmed, the size of the complete genome sequence was 523,056 bps larger than in the previous draft. These sequences were not clustered but scattered over the whole circular chromosome of 7,893,056 bp (Brito *et al.*, 2015). The complete genome of SBR5T contained 6,705 protein-coding genes and genes for 87 tRNAs and 27 rRNAs. While several gene annotations already offered interesting glimpses into the physiological potential, e.g., genes encoding different types of nitrogenases, catabolic enzymes for utilization of sugars, biosynthesis of vitamins, and antibiotics resistance, the most valuable asset of the complete genome sequence of *P. sonchi* genomovar Riograndensis SBR5T is that it provides a foundation for all systems-level analyses of this species (Brito *et al.*, 2015).

To gain insight into the evolution of the genus *Paenibacillus* on the genome level, the pangenome repertoire of *P. sonchi* was analyzed by using not only the complete genome sequence of the genomovar Riograndensis SBR5T (Brito et al., 2015), but also those of the genomovars Oryzarum CAR114, Oryzarum CAS34 and Sonchi LMG_24727 (Ribeiro et al. 2022). The genomovars shared 39% of all genes (4,365 of 11,205 genes). Next, a pangenome analysis of one of the five phylogenetically isolated clades of diazotrophic *Paenibacillus* genomes was performed since it comprised most *Paenibacillus* genomes harboring alternative nitrogenase genes (83 genomes passed the quality criteria) including that of *P. sonchi* genomovar Riograndensis SBR5T. About 1,100 genes make up this core pangenome (Ribeiro *et al.* 2022). The pangenome was determined to be open since an increasing tendency to expand the pangenome repertoire was observed if more genomes were incorporated, whereas the core pangenome was stabilized. This is characteristic of bacteria living in multiple or complex niches, and complex communities with larger effective population sizes (Ribeiro *et al.* 2022). The pangenome analysis helped the characterization of the occurrence of different types of nitrogenases in *P. sonchi* genomomar Riograndensis (s. below).

Furthermore, the genome of *P. sonchi* genomovar Riograndensis SBR5T served as the basis for a comprehensive RNAseq analysis that led to the determination of its RNA landscape (Brito et al., 2017a). That type of analysis demands the exposition of the studied organism to varied growth conditions, aiming to obtain RNA samples containing a wide diversity of expressed genes. To achieve such diversity, the bacterium underwent 15 different growth conditions that comprised temperature, pH, and nutrition variations, and the resulting RNA material was isolated and pooled in equal amounts for sequencing. Two different library generation methods were strategically applied, the first included the whole transcriptome of SBR5T and the second was prepared isolating only its primary transcripts with unaltered 5’-triphosphate ends. The native 5’-triphosphate ends are obtained by RNA hydrolysis followed by a treatment with a terminator 5′-phosphate-dependent exonuclease that digests all RNA having a 5′-monophosphate end but not the RNAs having a 5′-triphosphate end (Pfeifer-Sancar et al., 2013). That sort of library enabled a plethora of genetic characterization possibilities for SBR5T, such as the identification of conserved sequences of ribosome binding sites (RBS) and translation start motifs, transcription start sites (TSS), as well as the elements within the 5′ untranslated regions (5’UTRs) of genes including *cis*-regulatory structures. In combination, the two libraries helped identify novel transcripts, quantify transcripts abundance and detect operon structures in SBR5T. A total of 1,268 TSS were identified as belonging to the 5’UTRs of annotated genes and 1,082 belonged to novel transcripts. Most 5’UTRs were 25 to 50 bp long and those larger than 100 bp (209 of 1,268 analyzed) revealed a conserved aGGaGg RBS motif and ttgaca and TAtaaT for the -35 and -10 hexamer motives, respectively (Brito *et al.*, 2017a). The operon analysis unveiled a majority of monocistronic transcripts and a total of 622 operons and 248 sub-operons, the description of such operon structures in that study helped to understand the gene expression processes inherent to some PGPR traits in SBR5T, for example, its siderophore transport. Moreover, *cis*-regulatory RNA elements were identified using the Infernal tool (Nawrocki and Eddi, 2013), which resulted in the identification of the riboswitches present in the transcriptome of SBR5T, with further functional experiments showing a thiamine pyrophosphate (TPP) riboswitch interfering with the translational machinery within the thiamine biosynthesis of this bacterium (s. below) (Brito *et al.*, 2017a). Hence, the RNA landscape analysis provided a valuable basis for the development of genetic tools and omics-based characterizations such as differential RNAseq analysis of this bacterium.

**Figure 1.**
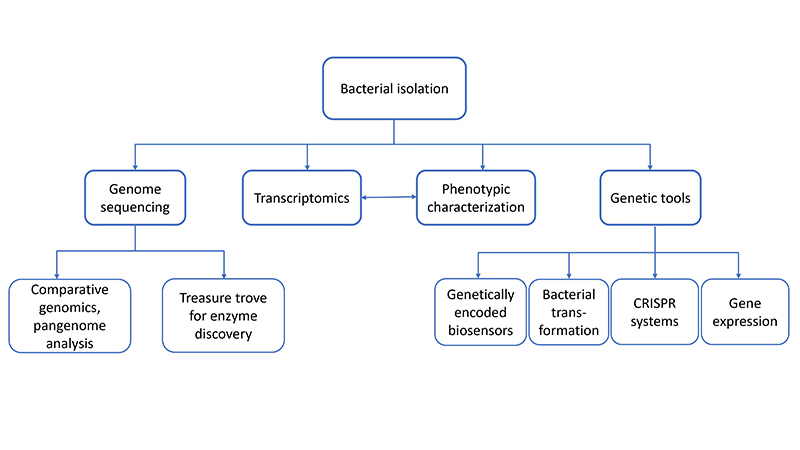
An exemplary route to omics-based characterization of a non-model microorganism. Analyses that were performed with the *P. sonchi* SBR5 strain and how they could be used to complement each other.

Figure 1 presents an overview of all studies that were done with *P. sonchi* SBR5 strain to contextualize how the analysis steps could be used together and how they might complement each other. Systems-level analyses of the proteome or the metabolome are still lacking but will be immensely valuable for further analysis of *P. sonchi*.

## *P. sonchi* genomovar Riograndensis SBR5T as a PGPR

### Nitrogen fixation

Biological nitrogen fixation (BNF), performed by diazotrophic organisms, can be considered the most important plant growth-promoting trait. The biosynthesis of reduced nitrogen from the inert dinitrogen gas in the Earth’s atmosphere by the process of BNF is catalyzed by nitrogenase enzymes. This reaction (EC 1.18.6.1) is very energy-demanding and requires 16 ATP and 4 reduced ferredoxins per fixed molecule of dinitrogen (Rubio and Ludden, 2008). Nitrogenase enzymes are comprised of two main subunits, dinitrogenase reductase and dinitrogenase. Due to their metal ion content, the subunits are named iron (Fe) protein and iron-molybdenum (FeMo) protein, respectively. In the catalytic cycle of nitrogen fixation, dinitrogenase enzyme directly reduces dinitrogen, whereas dinitrogenase reductase provides electrons for the reduction and energy by ATP hydrolysis (Hoffman et al., 2009). The FeMo-nitrogenase subunits are encoded in the operons *nifBHDKENXhesAnifV* and *nifENX*. In some diazotrophic bacteria, two additional nitrogenase enzymes can also be found: V-nitrogenase (EC 1.18.6.2) and Fe-only nitrogenase, which contain FeV and FeFe co-factors, respectively (Hu and Ribbe, 2015). Structural components of V-nitrogenase and Fe-only nitrogenase are encoded in the operons *vnfHDGK* and *anfHDGK* genes, respectively, plus an additional delta chain (encoded by *vnfG* or *anfG* genes, respectively) (Mus et al., 2018). Beyond the structural genes of the three types of nitrogenase enzymes, accessory genes responsible for co-factor cluster synthesis, nitrogenase assembly, electron transfer, and gene regulation are known. In *P. sonchi* SBR5T, besides the conventional FeMo-nitrogenase, a Fe-only nitrogenase was identified and demonstrated to be functional in the absence of Mo (Fernandes *et al.*, 2014).

Conventional nitrogenase (FeMo-nitrogenase) genes are regulated by GlnR in *P. sonchi* (Fernandes et al., 2017). This transcriptional regulatory protein represses transcription of the *nif* operon at high nitrogen status (Fernandes *et al.*, 2017). GlnR binding to its target DNA sequences was demonstrated by surface plasmon resonance spectroscopy. Importantly, the link to the nitrogen status of the cell was revealed since GlnR-DNA affinity was greatly enhanced when GlnR was bound by glutamine synthetase when the latter was feedback-inhibited due to binding glutamine. In addition, the energy status was monitored since complex formation between GlnR and glutamine synthetase depended on ATP and AMP levels within the cell. The complex is bound to multiple operator sites to form a loop for strong repression of its target genes (Fernandes *et al.*, 2017). While it was demonstrated that under Mo-limiting conditions the alternative Fe-only nitrogenase genes of *P. sonchi* are transcribed and catalytic activity of the Fe-only nitrogenase enzyme could be measured (Fernandes *et al.*, 2014), this type of regulation does not involve GlnR. The underlying regulatory mechanisms of nitrogen- and Mo-dependent regulation of the Fe-only nitrogenase enzyme remain elusive.

The evolution of the alternative nitrogenase has been studied in some detail in the Paenibacillaceae family by bioinformatics analysis (Ribeiro et al., 2022). Of 930 genomes in the Paenibacillaceae family, 160 were identified as putative diazotrophic genomes. Thus, 17% of these species are expected to fix nitrogen. Of these, only a subset making up 2.5% of all Paenibacillaceae genomes possess genes for the alternative Fe- or V- nitrogenase enzymes (Ribeiro *et al.*, 2022). Genomes encoding the Fe-only nitrogenase shared two operons, *nifEN* and *anfHDGK*, and belonged to the three genera *Gorillibacterium, Fontibacillus,* and *Paenibacillus,* in the latter there is the addition of the *nifX* in the operon that contains the *nifEN* genes. The species phylogeny of Paenibacillaceae separated the diazotrophs into five clades, one of these containing all occurrences of strains harboring alternative nitrogenases in the *Paenibacillus* genus. It was proposed that Fe-nitrogenase was acquired by the ancestral lineage of the genera *Fontibacillus, Gorillibacterium,* and *Paenibacillus* via horizontal gene transfer (Ribeiro *et al.*, 2022). Later, gene transfers and gene losses shaped the evolution of the alternative nitrogenases in these groups, and accessory genes may have coevolved. Taken together, the trait of nitrogen fixation is sparse within the Paenibacillaceae and the presence of Fe-only nitrogenase in the *P. sonchi* group was exclusive to the genomovar Riograndensis (Ribeiro *et al.*, 2022). Thus, the insight gained about the Fe-only nitrogenase in the *P. sonchi* group revealed the importance of establishing omics-based analysis to further our understanding of the evolution of rare metabolic traits in non-model microorganisms.

### Iron acquisition

Besides nitrogen fixation, the production of siderophores is highly relevant for promoting plant growth. Siderophores complex iron ions and enable bacteria to access iron under iron-limiting conditions. An RNAseq analysis of *P. sonchi* in response to iron depletion revealed that RNA levels were increased for 71 genes and decreased for 79 genes (Sperb et al., 2016). Besides gene expression changes related more generally to impaired growth like sporulation and DNA protection genes, the gene *fecE* encoding a Fe^3+^ siderophore transporter was upregulated. Indeed, the FecE transporter has presented high expression in the SBR5T landscape transcriptome as well (Brito et al., 2017a). However, although it was demonstrated that SBR5T can produce siderophores (Beneduzi et al., 2010), genes related to siderophore biosynthesis were not induced under iron starvation conditions (Sperb *et al.*, 2016).

### Phosphate solubilization

Phosphorus (P) is an essential nutrient for plant development. While inorganic phosphate for use as fertilizer currently is extracted from P-rich rock and its world supply is finite, P solubilization by PGPR is an important consideration. About half of the roughly 4.5 million tons of P used as fertilizer are lost by soil immobilization and surface runoff (Granada et al., 2018). The authors calculated that PGPR may provide as much as 0.8 million tons of P to plants, thus, reducing the requirement for inorganic phosphate as fertilizer by one-third (Granada *et al.*, 2018).

*P. sonchi* can solubilize hydroxyapatite, generating 1 mM free P from that insoluble P source in shake flask conditions. Differential gene expression analysis compared growth with P provided as soluble P (NaH_2_PO_4_) or as insoluble P (hydroxyapatite). The RNAseq analysis determined that RNA levels of 68 genes were increased during growth with insoluble P and 100 genes were down-regulated. To reach these results, first, the sequence reads were mapped onto the reference genome of *P. sonchi* SBR5 (Brito et al., 2015). Then, the tool Trimmotatic version 0.33 (Bolger et al., 2014) was used to trim the sequences to a minimal length of 35 bps. These reads were mapped to SBR5 genome through the software Bowtie (Langmead, 2010). The software ReadXplorer (Hilker et al., 2014) was then used for the visualization of mapped reads and to perform the differential gene expression analysis. The statistical method DEseq (Anders and Huber, 2010) was employed to analyze the resultant RNAseq data. To classify as differentially expressed, the gene needs to have a change in expression level higher than 30 and a P-value equal to or less than 0.05. Quantification of metabolites in the culture broth revealed higher concentrations of the osmolytes proline, trehalose, and glycine betaine during growth with insoluble P as compared to soluble P (Brito *et al.*, 2020a). Moreover, the osmolality levels in both intracellular and extracellular contents under the hydroxyapatite cultivation were superior to that under the soluble P cultivation. Accordingly, RNA levels were increased for glycine betaine uptake genes *opuAA* and *opuAB* as well as for the biosynthesis of proline (*proC*) and trehalose (*treA*) (Brito *et al.*, 2020a). In the same study, a promoter-reporter gene fusion assay showed that the promoter belonging to the first gene in the glycine betaine transporter operon, *opuAA*, was induced solely under insoluble P cultivation, in contrast to soluble P condition. That reinforced the idea that *P. sonchi* SBR5 invests in osmoprotection when solubilizing phosphates. Growth with insoluble P was also characterized by reduced RNA levels of TCA cycle genes *odhAB* and reduced secretion of TCA cycle-derived organic acids. Certainly, under the conditions of insoluble P, the specific activity of the SBR5T *odhAB*-encoded enzyme 2-oxoglutarate dehydrogenase was entirely depleted, whereas it remained active in the presence of soluble P. By contrast, *P. sonchi* secreted gluconic acid and acetic acid to solubilize insoluble P, indicating the secretion of organic acids as the main strategy used by this organism for phosphate solubilization. Furthermore, the expression of motility genes was reduced and those of thiamine biosynthesis were increased during growth with insoluble P (Brito *et al.*, 2020a). This comprehensive analysis is the first step and basis to gain a deeper insight into P solubilization by *P. sonchi* and has the potential to further improve its use as a crop inoculant regarding the provision of soluble P to plants.

### Other traits

The analysis of *P. sonchi* regarding its plant-growth-promoting traits nitrogen fixation, siderophore production, and phosphate solubilization need to be complemented by further characterizations, e.g., with respect to producing plant hormones and antibiotics, but also to define its response to interaction with plants or pathogens. Moreover, most analysis performed to date combined RNAseq analysis with physiological characterization, but did not yet include functional analysis of genes by gene overexpression (gain-of-function) or repression/deletion (loss-of-function).

## Genetic tools for functional gene analysis in *P. sonchi*

### Transformation

**Figure 2.**
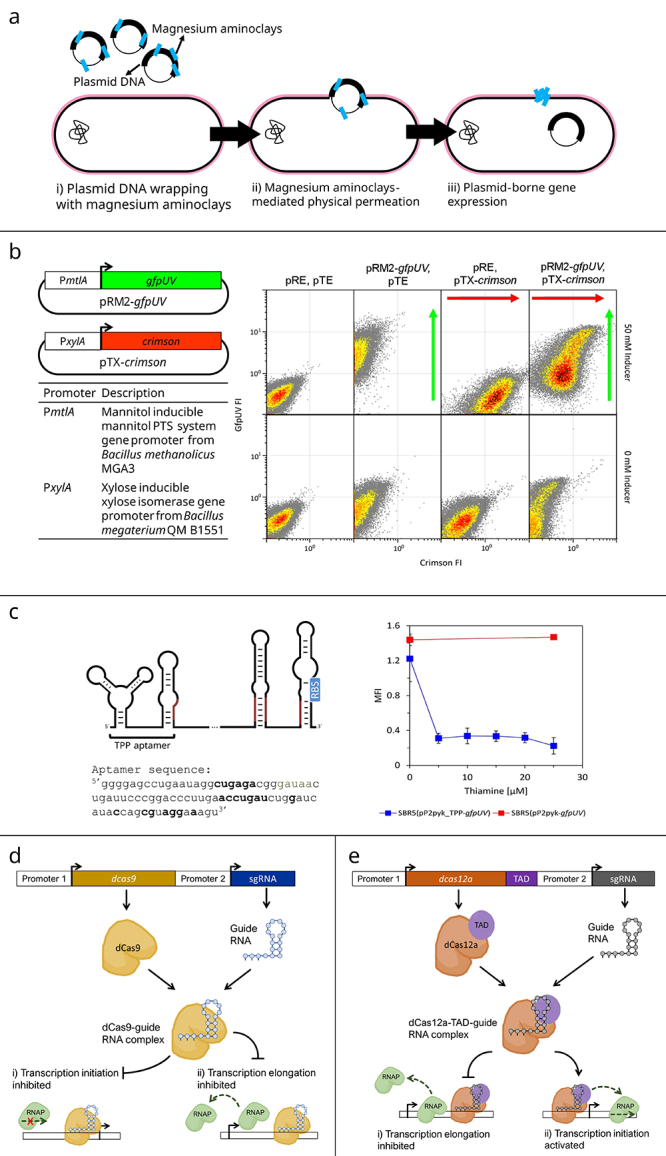
Genetic tools useful for application in *Paenibacillus* functional gene analysis. a) Schematic representation of the magnesium aminoclay-based genetic transformation, the so-called Yoshida effect transformation, that involves wrapping DNA with aminoclay and cell membrane physical permeation by means of friction power. **b)** Flow cytometry analysis of reporter gene expression in *P. sonchi* genomovar Riograndensis SBR5T cells under four different conditions: carrying the compatible rolling circle- and theta-replicating plasmids (pR and pT, respectively), and either with or without inducers xylose and mannitol. Adapted from Brito et al. (2017b). **c)** Schematic view of the *P. sonchi* TPP riboswitch (left) and the influence of the TPP riboswitch on GfpUV fluorescence intensity (MFI) in *P. sonchi* cells under increasing thiamine concentrations. The expression of *gfpUV* was controlled by thiamine levels using two different promoters: PpyK with the 5’ UTR replaced by the *thiC* gene’s 5’ UTR, and the other using the native 5’ UTR of the *pyk* promoter. Adapted from Brito *et al.* (2017a). **d)** Schematic view of CRISPRi gene repression by competition with RNA polymerase (RNAP) or transcription factors in transcription initiation or by blocking transcription elongation. **e)** Dual effect of CRISPRi/a by fusing the *dcas12a* to a transcription activator domain (TAD).

Genetic engineering for gain- and loss-of-function analysis is important to study genotype-phenotype relations, thus, genetic transformation has to be established, which often poses a challenge for non-model microorganisms. *P. sonchi* is difficult to transform by electroporation (Bach et al., 2016a), but a rarely used method to transform bacteria was shown to be applicable to this bacterium, namely physical permeation by magnesium amino-clays (Brito et al., 2017b). This method differs from chemical, electro, biolistic, or sonic transformation, as it relies on the Yoshida effect (Yoshida et al., 2001). A colloidal solution with a nanosized acicular material is applied to exogenous DNA and bacterial cells to increase the frictional coefficient rapidly. Upon penetration of the resulting large complex into bacterial cells, exogenous DNA is taken up into the cells (Figure 2a) (Yoshida and Sato 2009). Transformation of *P. sonchi* was shown by plasmid isolation and re-transformation as well as by heterologous production of three different fluorescent reporter proteins (Crimson, GFPuv, and mCherry). The protocols and plasmids could be transferred to *P. polymyxa* DSM365 (Brito *et al.*, 2017b).

### Plasmids for constitutive and inducible gene expression

A suite of cloning and expression vectors with different modes of replication as well as for constitutive or inducible gene expression was developed for functional gene characterization and stable genetic engineering. Two compatible plasmids were designed for *P. sonchi*, one of which was based on the *Staphylococcus aureus* rolling circle-replicating vector pNW33N (pC194), while the other was based on the theta-replicating pHCMC04 (pBS72) from *Bacillus subtilis*. The latter supports higher genetic stability, while the former provides higher gene dosage. Both were shuttle vectors for *Escherichia coli*, the preferred DNA cloning host (Brito et al., 2017b).

To enable constitutive expression, three promoters were chosen as they were known to be well expressed in other bacteria: the endogenous promoters of the EF-TU gene *tuf*, of the glyceraldehyde 3-phosphate dehydrogenase gene *gap*, and of pyruvate kinase gene *pyk*. When the promoters were fused to the promoter-less *gfpUV* gene and cloned into the rolling circle-replicating vector and used to transform *P. sonchi* genomovar Riograndensis SBR5T and *P. polymyxa* DSM365. Although the absolute GfpUV fluorescence values differed, in both *Paenibacillus* species the rank order of the promoter strengths was Pgap > Ptuf > Ppyk (Brito et al., 2017b).

To enable inducible gene expression, two systems were used: the xylose-inducible XylR regulation system from *Bacillus megaterium* (PxylA) and the mannitol-inducible systems from *B. subtilis* and *Bacillus methanolicus* (PmtlA). The xylose-inducible system was shown to work well with the theta-replicating and the rolling circle-replicating vectors. The mannitol-inducible system was functional with the respective promoters from *P. sonchi* and *B. methanolicus*, but not from *B. subtilis* (Brito et al., 2017b).

The compatible vectors pRM2-*gfpUV* and pTX-*crimson*, with mannitol- and xylose-inducible systems, respectively, were used consecutively to transform *P. sonchi* genomovar Riograndensis SBR5T and the recombinant SBR5(pRM2-*gfpUV*)(pTX-*crimson*) was cultivated either without inducers, with 50 mM xylose alone, with 50 mM mannitol alone and with a mixture of 50 mM xylose and 50 mannitol (Figure 2b). As expected, in the presence of only one inducer single-fluorescence-positive cells were observed by flow cytometry, while double-fluorescence-positive cells were only observed in the presence of both inducers (Figure 2b) (Brito et al., 2017b). The system could be transferred to *P. polymyxa* DSM365 (Brito *et al.*, 2017b).

**Figure 3.**
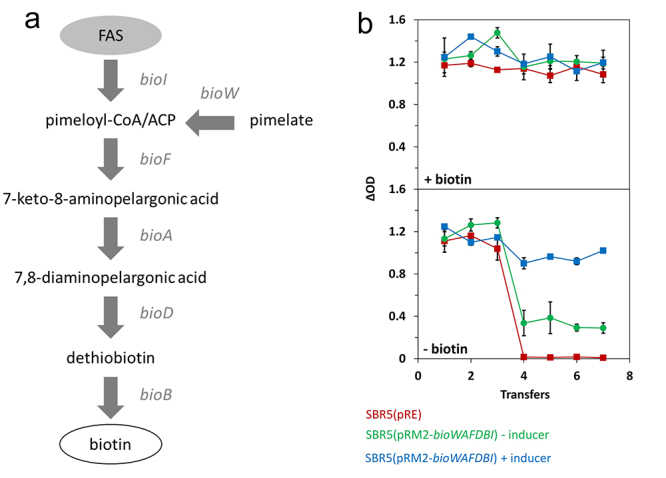
Example of how to apply the genetic tools developed to demonstrate a genetic cause deduced form from the genome sequence for an observed phenotype (here, biotin auxotrophy). The biotin requiring *P. sonchi* genomovar Riograndensis SBR5T was converted to a biotin-prototrophic strain based on mannitol-inducible expression of the biotin biosynthesis operon *bioWAFDBI* from *B. subtilis*. a) Schematic view of biotin biosynthesis in *B. subtilis*. b) Growth of *P. sonchi* empty vector strain SBR5(pRE) (red squares) and the expression strain SBR5(pRM2-*bioWAFDBI*) in minimal medium lacking or containing biotin. SBR5(pRM2-*bioWAFDBI*) was cultivated without (green squares) or with induction with mannitol (blue squares). The biomass values (**Δ**OD) represent growth after repeated transfers to fresh medium. Figure adapted from Brito et al. (2017b).

As an example of a metabolic engineering application, the biotin auxotrophic *P. sonchi* genomovar Riograndensis SBR5T was converted to a biotin-prototroph when the biotin biosynthesis operon *bioWAFDBI* from *B. subtilis* was expressed using the mannitol-inducible expression system. The recombinant SBR5(pRM2-*bioWAFDBI*) grew stably for seven serial transfers to a biotin-free medium when gene expression was induced by mannitol, but not uninduced (Figure 3) (Brito et al., 2017b).

### CRISPRi

To obtain valuable insights regarding gene function in *P. sonchi* genomovar Riograndensis SBR5T, the available genetic toolbox was expanded beyond gene expression. The established genetic tool was based on CRISPR (clustered regularly interspaced short palindromic repeats) technology. Many review papers on synthetic biology suggest that CRISPR technology has revolutionized the field by providing a precise and efficient way to regulate and modify microbial metabolism (Cleto et al., 2016; Kim et al., 2017; Wang et al., 2019; Schultenkämper et al., 2020; Zhan et al., 2020). This way, CRISPR technology allows the targeted manipulation of metabolic pathways in PGPR towards gain- or loss-of-function characterization of plant growth-promoting features. CRISPR is a highly precise gene editing technology that allows modifications of DNA sequences. It uses a single-stranded guide RNA (sgRNA) complexed with a Cas enzyme to target specific genes and to cut and edit the DNA at that location. Based on the same technology, CRISPR interference (CRISPRi) is a tool that uses an endonuclease-deactivated Cas enzyme (dCas) that binds to DNA and blocks gene expression without cleaving the DNA sequence (Figure 2d). Furthermore, when fusing dCas with a transcriptional activator, dual mode control becomes possible to either activate (CRISPRa) or repress gene expression (Figure 2e) (Schultenkämper *et al.,* 2020).

Besides *P. sonchi*, *Paenibacillus* species have been the subject of several studies involving the aforementioned variants of CRISPR technology. Rütering et al. (2017) successfully created a pUB110-derived CRISPR-Cas9 vector system for genome editing in *P. polymyxa* DSM 365. The system was applied to elucidate and increase the biosynthesis of a wide range of exopolysaccharides in that organism (Rütering *et al.* 2017; Schilling et al., 2022; Schilling *et al.,* 2023). Furthermore, the same system was adapted in two approaches: homology-directed repair in large gene clusters targeted by the Cas9-sgRNA system; and the combined use of multiple sgRNAs towards multiplexed gene deletions and insertions in *P. polymyxa* (Meliawati et al., 2022). The first approach resulted in the deletion of gene clusters related to exopolysaccharides and antibiotic production (12-41 kb), while the multiplex deletion was achieved with more than 80% efficiency.

While CRISPR, CRISPRi and CRISPRa use the same basic components, they have different applications. CRISPR is typically used in metabolic engineering to introduce permanent genetic changes such as deletions or SNPs, while CRISPRi and CRISPRa are commonly used to study gene function and characterization by knockdown and overexpression. A novel CRISPRa technology based on a dCas12a linked to the transcription activator (TA) SoxS was developed for multiplexed gene expression activation in *P. polymyxa* (Schilling et al., 2020). Gene activation takes place upstream of the targeted gene by RNA polymerase recruitment. The same system allows gene repression simultaneously with activation, when additional sgRNAs target genomic regions within open reading frames blocking transcription elongation (Figure 2e) (Schilling *et al.,* 2020). In *P. sonchi*, a pNW33N plasmid-based CRISPRi tool developed for *B. methanolicus* by Schultenkämper et al. (2019) was used as a basis for establishing gene repression (Brito et al., 2020b). The CRISPRi system used dCas9-sgRNA system to target endogenous sporulation genes *spo0A*, *yaaT*, and a sorbitol dehydrogenase gene *ydjJ*. By using CRISPRi-based *spo0A* and *yaaT* repression, it was observed that sporulation decreased, while biofilm formation increased in *P. sonchi*. The repression of *ydjJ* resulted in decrease in specific activity of sorbitol dehydrogenase in crude cell extracts and reduced biomass formation from sorbitol in shake flask cultivation (Brito *et al.*, 2020b). While the chosen gene targets served to demonstrate the repression of regulatory genes (with low expression) as well as metabolic genes (with high expression) by CRISPRi technology, the experiments demonstrated the functional roles of *spo0A*, *yaaT*, and *ydjJ* for sporulation, biofilm formation and carbon source utilization.

Notably, the genetic tools for gene expression developed for *Paenibacillus* species could readily be transferred between *P. sonchi* and *P. polymyxa* (Brito et al., 2017b). Therefore, it is very plausible to explore and adapt the CRISPR systems developed for *P. polymyxa* for use in *P. sonchi*’s future research.

### Genetically encoded biosensor

Monitoring intracellular metabolite concentrations in single bacterial cells is challenging, but genetically encoded biosensors have been developed to provide ample opportunity to determine the physiologically relevant intracellular concentration ranges and to study intracellular metabolite concentrations at the single-cell level, e.g., by cytometry. The genetically encoded biosensors typically are based on a transcriptional regulator protein that senses the metabolite as an inducer or coactivator and controls a fluorescent reporter gene via its target promotor. A recently developed class of genetically encoded biosensors is based on riboswitches. The aforementioned RNA landscape analysis of *P. sonchi* identified *cis*-regulatory elements in the 5’UTRs, among them a TPP-dependent riboswitch as part of the thiamine biosynthesis gene *thiC* (Brito et al., 2017a). The 5’UTR of *thiC* including the TPP riboswitch was cloned between a constitutive promoter and the open reading frame of *gfpUV* such that transcription of *gfpUV* was expected to be constitutive, while translation initiation was expected to respond to different intracellular TPP concentrations. The functionality was shown when GFPuv fluorescence quantified by flow cytometry was low in the presence of externally added thiamine, while it was about fourfold higher in the absence of added thiamine (Figure 3) (Brito *et al.,* 2017a). Thus, the biosensor based on the TPP riboswitch can be used to monitor intracellular TPP concentrations.

The RNAseq analysis comparing growth with soluble P and insoluble P revealed that RNA levels of genes of thiamine biosynthesis were increased when only insoluble P was available (Brito et al., 2020a). The TPP biosensor was then used to verify that indeed intracellular TPP levels differed. The TPP biosensor fluorescence was high during growth with soluble P, but low during growth with insoluble P, which indicated that thiamine biosynthesis occurred during growth with insoluble P (Brito *et al.*, 2020a).

### Learning from *P. sonchi*: a source of enzymes

Biotechnological production by fermentation, whole-cell transformation, enzyme catalysis, or bio-chemo-catalysis requires specific enzymes that operate isolated, cascaded, or in (synthetic) metabolic pathways. Here, one of the central quests is the identification of the specific enzyme. Nature provides a large source for enzymes that has been complemented by enzyme evolution to enable new-to-nature chemistry (Nobel Prize in Chemistry, 2018, to Frances Arnold). To find new or better microbial enzymes in nature, microbes with large genomes are key since these do not only rely on a minimal gene set for survival but are equipped with a large metabolic potential to cope with complex and changing habitats. In this respect, *P. sonchi* genomovar Riograndensis is a rich source of biological functions since it is striving in a varied habitat and, thus, has a large genome with 6,705 protein-coding genes (Brito et al., 2015). Most of the genes have been annotated, but only a few have been characterized by genetic or biochemical means. Nonetheless, the genome sequence is a treasure trove for recombinant applications in which *P. sonchi* is the donor and other bacteria such as *E. coli* or *Corynebacterium glutamicum* are the acceptors.

*P. sonchi* showed activity for acid phosphatase, C4 esterase, α-glucosidase, β-galactosidase and was shown to degrade glycerol, malate, raffinose, *N*-acetylglucosamine, mannitol, mannose, arabinose and starch (Beneduzi et al., 2010; Siddiqi et al., 2017). Three genes for putatively starch-degrading enzymes have been heterologously expressed in *C. glutamicum*, a bacterium used in the biotech industry for the million ton per year amino acid production (Wendisch, 2020). *C. glutamicum* can utilize several carbon sources but has to be engineered to enable access for others (Wendisch *et al.*, 2016). While the species description of *P. sonchi* showed that it can hydrolyze starch (Beneduzi *et al.*, 2010), *C. glutamicum* cannot. Expression of the *P. sonchi* genes PRIO_3240 and PRIO_423 coding for putative α-amylase and α-amylopullulanase enzymes in the starch-negative *C. glutamicum* enabled the recombinant to grow with starch (Brito, Walter, and Wendisch, *unpublished*) as shown also for the amylase gene of *Streptomyces griseus* (Sgobba et al., 2018). More generally, other *Paenibacillus* species may extend the application potential of biogenic polymers and their uses. For example, *P. xylanilyticus* is a mesophilic, facultative, anaerobic, xylanolytic, and cellulolytic bacterium, and corresponding to its habitat a rich source of polysaccharide degrading enzymes. Cellulose is degraded involving lytic polysaccharide monooxygenases (Ito et al., 2023), xylans by xylosidases (Ito *et al.*, 2022), chitin by chitinases, chitosanases and β-*N*-acetylhexosaminidases, alginate by alginate lyases (Itoh, 2021), and pectin by pectin methylesterases and lyases (Zhong et al., 2021). In this respect, the full potential of *P. sonchi* is yet to be tapped.

**Figure 4.**
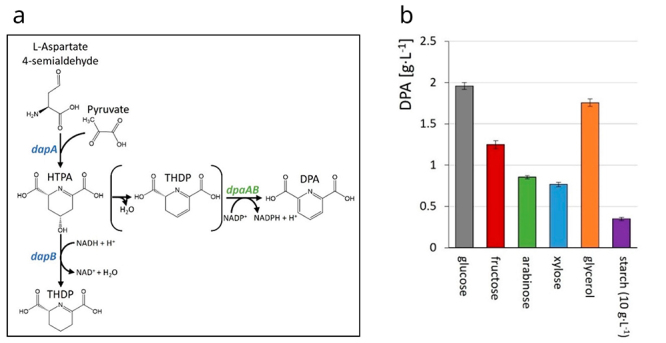
Example for having tapped the genome sequence to discover an enzyme and to apply this enzyme in an industrially relevant biotechnological application. Dipicolinic acid (DPA) production by recombinant *C. glutamicum* expressing DPA synthase genes *dpaAB* from *P. sonchi*. a) Scheme of the bifurcation of DPA biosynthesis from the L-lysine biosynthetic pathway. b) Production of DPA by engineered *C. glutamicum* from glucose, arabinose, xylose, glycerol and starch with glucose as co-substrate. Abbreviations: HTPA synthase gene (*dapA*); dihydrodipicolinate reductase gene (*dapB*); hydroxy-2,3,4,5-tetrahydrodipicolinic acid (HTPA); 2,3,4,5-tetrahydrodipicolinic acid (THDP). Figure adapted from Schwardmann *et al.* (2020).

*P. sonchi* genomovar Riograndensis was also the source for genes of dipicolinic acid (DPA) biosynthesis (Figure 4a). In nature, DPA occurs in endospores of Gram-positive bacteria, in particular anaerobic *Clostridum* and aerobic *Bacillus* species. Sporulation initiates under adverse conditions and DPA biosynthesis is involved in this process. In the endospore, DPA chelates calcium ions and accumulates to about 10% weight of the *B. subtilis* endospore to prevent DNA denaturation and mediate heat resistance of the endospore (Schwardmann et al., 2022). Although DPA has not been quantified in *P. sonchi*, the genes encoding the subunits of the enzyme dipicolinate synthase from this bacterium were heterologously expressed in *C. glutamicum* strains overproducing precursors of L-lysine to establish the first *de novo* production of DPA by *C. glutamicum* (Schwardmann *et al.*, 2022) (Figure 4b). The intermediate of L-lysine biosynthesis 4-hydroxy-tetrahydrodipicolinate (HTPA) was converted by dipicolinate synthase DpaAB to DPA by dehydration and oxidation to yield the heterocyclic aromatic dicarboxylic acid DPA. Since heterologous expression of the *P. sonchi* genes was required for this enzymatic activity, they were demonstrated to code for functional dipicolinate synthase. DPA is non-toxic, easily biodegradable, heat-stabile, and has many technical applications ranging from metal chelation, as an antimicrobial, antioxidant, for production of pyridines and piperidines, stabilization of peroxides, and as the monomeric precursor of biopolymers. Since *C. glutamicum* was engineered to utilize various second-generation feedstocks, the production of DPA using the recombinant *C. glutamicum* strain with the *P. sonchi dpaAB* genes could be based on renewable carbon sources such as the pentose sugars xylose and arabinose and was shown to operate up to the 2.5 L bioreactor scale (Schwardmann *et al.*, 2022).

## Conclusion

As was demonstrated in this review, we have learned a lot about *P. sonchi* genomovar Riograndensis SBR5T, showing how we can explore strains isolated from the environment through various methodologies. In one hand, it has been studied regarding the regulation and evolution of nitrogen fixation systems, regarding the alternative Fe-only nitrogenase. On the other hand, genetic tools including CRISPRi have been developed to perform functional gene analysis and applied to gain insight into its physiology such as vitamin biosynthesis. Genome, pangenome, and transcriptome analyses laid the foundation to explore the large genome of SBR5T as a rich source of biological functions highlighting its potential in providing environmental and economic benefits.
